# Characterization of bovine miRNAs by sequencing and bioinformatics analysis

**DOI:** 10.1186/1471-2199-10-90

**Published:** 2009-09-16

**Authors:** Weiwu Jin, Jason R Grant, Paul Stothard, Stephen S Moore, Le Luo Guan

**Affiliations:** 1 Department of Agricultural, Food and Nutritional Science, University of Alberta, Edmonton, AB T6G2P5, Canada

## Abstract

**Background:**

MicroRNAs (miRNAs) are a family of ~22 nucleotide small RNA molecules which regulate gene expression by fully or partially binding to their complementary sequences in mRNAs or promoters. A large number of miRNAs and their expression patterns have been reported in human, mouse and rat. However, miRNAs and their expression patterns in live stock species such as beef cattle are not well studied.

**Results:**

We constructed and sequenced small-RNA libraries to yield a total of 13,541 small-RNA sequences from 11 bovine tissues including brain, subcutaneous fat, muscle, liver, kidney, spleen and thymus. In total, 228 miRNAs including 29 novel miRNA candidates were identified. Of the 199 miRNAs, 101 have been previously reported as bovine miRNAs and the other 98 are bovine orthologs of known miRNAs that have been identified in at least one other mammalian species. Of the 29 novel miRNA candidates, 17 appeared at this point in time to be bovine specific, while the remaining 12 had evidence of evolutionary conservation in other mammalian species. Five miRNAs (miR-23a, -23b, -99a, -125b and -126-5p) were very abundant across the 11 tissues, accounting for 44.3% of all small RNA sequences. The expression analysis of selected miRNAs using qRT-PCR also showed that miR-26a and -99a were highly expressed in all tissues, while miR-122 and miR-133a were predominantly expressed in liver and muscle, respectively.

**Conclusion:**

The miRNA expression patterns among 11 tissues from beef cattle revealed that most miRNAs were ubiquitously expressed in all tissues, while only a few miRNAs were tissue specific. Only 60% miRNAs in this study were found to display strand bias, suggesting that there are some key factors for mature miRNA selection other than internal stability. Most bovine miRNAs are highly conserved in other three mammalian species, indicating that these miRNAs may have a role in different species that are potential molecular markers for evolution.

## Background

MicroRNAs (miRNAs) are small non-coding RNA molecules of approximately 22 nucleotides in length, which play important regulatory roles in animals and plants [1,2]. miRNAs have been found to down-regulate the expression of target genes by binding to the complementary sites in transcripts and causing translational repression or transcript degradation [3]. Numerous biological processes in animal development, apoptosis, fat metabolism and hematopoietic differentiation have been reported to be regulated by miRNAs [4-7]. In addition, recent studies have revealed that miRNAs can increase protein translation by binding to complementary promoter sequences, extending the important function of miRNA to protein expression [8,9].

Many experimental techniques and computational methods have been developed to identify miRNAs [10-12]; and a large number of miRNAs have been identified in primates, rodents, birds, fish and plants [13-16]. However, the number of miRNAs from bovine species is limited with only 125 reported (miRBase 12.0, December 2008), comparing with 866 from human and 627 from mouse. Early studies suggested that most miRNAs are conserved among related species [17,18]. However, recent studies have shown that many newly identified miRNAs are species specific [19], and the expression of miRNAs is not strictly conserved among species [20]. Although many miRNAs are known to be differentially expressed during development and across tissue types [4-6,21], little is known about the relative abundance and specificity of expression patterns among tissues for most bovine miRNAs. In this study, we profiled bovine miRNAs and evaluated their expression patterns from 11 beef cattle tissues including muscle, kidney, liver, spleen, thymus, three fat tissues and three brain tissues. Elucidation of the expression patterns of different miRNAs among different tissues will contribute to the understanding of the roles of miRNAs in gene expression regulatory networks for particular biological functions in livestock species.

## Results

### Identification of miRNAs and novel miRNA candidates

From 11 small RNA libraries, 11,880 clones were sequenced and 13,541 small RNA sequences were obtained with 1,000 to 1,400 small RNA sequences covered from each library. Among the total of 12,869 sequences, 11,189 sequences were classified as previously reported bovine miRNAs, 1,578 sequences were orthologs of known miRNAs from other mammalian species, and 102 sequences were novel miRNA candidates. The rest sequences were found to be other small non-coding RNA, mRNA, tRNA or rRNA, *etc*. (Table 1, Additional file 1).

**Table 1:** An overview of sequencing results from 11 small RNA libraries

	**Cer**	**Hyp**	**Med**	**LDM**	**Asf**	**Bsf**	**Rsf**	**Liv**	**Kid**	**Spl**	**Thy**	**Total**
Sequences of reported bovine miRNAs	975	786	927	488	1238	1089	1308	1267	1080	882	1149	11189
Sequences of orthologs of known miRNAs	144	140	160	492	81	67	43	50	82	220	99	1578
Sequences of novel miRNA candidates	6	8	6	1	10	5	13	8	16	6	23	102
Sequences of other small RNAs	37	66	139	62	46	51	24	71	53	84	39	672
Overall sequences	1162	1000	1232	1043	1375	1212	1388	1396	1231	1192	1310	13541
Reported bovine miRNAs	62	46	47	44	57	49	42	54	66	60	68	101
Orthologs of known miRNAs	39	30	31	26	20	14	17	18	33	29	30	98
Novel miRNA candidates	6	5	6	1	3	3	4	3	6	4	3	29
Overall miRNAs	107	81	84	71	80	66	63	75	105	93	101	228

Eleven tissues from beef cattle were used for libraries construction. Library was named as abbreviated tissue name (detail see abbreviation).

**Figure 1:**
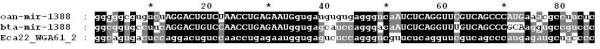
**Conserved mir-1388 in three mammalian species**. Mature miRNA sequences from bovine (bta) and platypus (oan) are showed in uppercase. Future name is shown as record ID for horse orthologs of mir-1388. Identical base is labeled in black or grey.

In total, we identified 101 previously reported bovine miRNAs and 98 orthologs of known miRNAs from other mammalian species, which are predicted to be encoded by 191 different miRNA genes (Additional file 2). In all libraries but longissimus dorsi muscle (LDM) library, the previously reported bovine miRNA sequences represented ~75% to 95% of the total sequences of each library. Nine out of 101 known bovine miRNAs and 23 out of 98 orthologous miRNAs were found to be present only once in the total sequenced clones (Additional file 3). Orthologs of known miRNAs sequences from LDM library accounted for approximately one third of all orthologous miRNAs (Table 1). Two orthologous miRNAs (bta-miR-574-3p and -652) were detected more than two times and in at least two different tissues but were not mapped to the bovine genome (ucsc_btau4) (Additional file 3). All but two of the bovine orthologous miRNAs had orthologs in human species. These two miRNAs were found to be produced from different arms of the same pre-miRNA (bta-mir-1388) and conserved in platypus and horse (Figure 1). In addition, we identified 29 novel miRNA candidates with 22 of them found to be present only once in all sequenced clones (Table 2).

**Table 2:** Novel miRNA candidates identified in this study

**ID**	**Sequence^*a*^**	**Genomic location**	**Strand**	**Number^*b*^**	**Conservation^*c*^**
bta-un01	AAAAACCUGAGUGAACUUUUC	chr16:7583053-7583073 chr27:21096115-21096135	- +	1	
bta-un02	AAACAUCUGGUUGGUUGAGAGA	chrX:3009839-3009860	-	1	D; E
bta-un03	AAACCCGAACGAACUUUUGGGCC	chr5:124162584-124162606 chr12:31995980-31996002 chr24:34630276-34630298 chr24:42515045-42515067	- - + +	1	
bta-un04	AAAGCUGAAUGAACUUUUUGGC	chr14:58301736-58301757 chrX:291566-291587 chrUn.004.21:72057047-72057068	+ + +	1	
bta-un05	UUUGCACCUCUGAGAGUGGAG	chr7:49895499-49895519	+	1	H; C; E
bta-un06	AACCCCAGAGAAACUUUCUGGC	chr8:43938121-43938142	-	1	
bta-un07	AAGAGUUUGUUCGGGUUUCUC	chrUn.004.13:739780-739800	+	2	
bta-un08	AAGUACAGGAUGCCCAAUGAAU	chr21:65541270-65541291	-	1	
bta-un09	AGCCCUUCCCUUUCACUGGCCU	chr28:32693949-32693969	-	3	E
bta-un10	AGCGAGGUUGCCCUUUGUAUAU*	chr21:66021949-66021970	+	1	H; M; R; C; E
bta-un11	CAGUCCGGUCCCGCGGUGUCUCC	chr1:76369850-76369872	+	1	
bta-un12	CCUCAGUCAGCCUUGUGGAUGU	chrX:21829475-21829496	-	56	H; E
bta-un13	GAAGUUGCCCAUGUUCUUUUCG*	chr21:66012119-66012140	+	1	H; M; R; C; E
bta-un14	GAGAGAUCAGAGGCGCAGAGU	chr1:65888852-65888872	-	2	H; C; E
bta-un15	GCGGCCCGCGGGCUCGGAUGCUA	chr1:76369888-76369908	+	1	
bta-un16	UUACUCUGAGUAACCUAACUGU	chr26:31723064-31723084	+	1	
bta-un17	GGACUUCCCUGGUAGCUCAGC	chrX:18300110-18300129 chrX:82012989-82013008	+ -	2	
bta-un18	GGUUGAUCAGAGAACAUACAUU	chr21:66028357-66028378	+	1	H; C; E
bta-un19	GUCAGGAUGGCCGAGUGGUC	chr7:42028223-42028242	+	1	
bta-un20	GUGGUUAUCCCUGCUGUGUUCG*	chr21:66028745-66028766	+	1	H; C; E
bta-un21	UCCCCUUCCUUCCGGCCUCCGCC	chr21:68561367-68561389	+	1	
bta-un22	UCCGCCUUGUGCUUCCUGCAU	chr11:102046802-102046821	+	1	
bta-un23	UGAAAAGUUCGUUCGGGUUUUU	chr1:3474029-3474049	-	9	
bta-un24	UGCGGGAUCUUUAGUUGUGGUG	chr21:55700383-55700404 chr25:35972465-35972486	- -	6	
bta-un25	UGGGGGGAGGCCACACCAUGUCA	chr7:5546190-5546212	-	1	
bta-un26	UUGUGGCCGCAGCAACCUCGGU	chr10:66855690-66855711 chr10:66857969-66857990	- +	1	H; M; R; C; E
bta-un27	UUCGACUCCCGGUGUGGGAACCA	chr12:14082775-14082795 chrUn.004.4221:9671-9691	- +	1	H; M; R; C; E; G
bta-un28	GUGGACUUCCCUGGUAGCUCAGCU	chr4:106953595-106953618	+	1	
bta-un29	GUCAGGAUGGCCGAGCGGUCU	chr3:8630409-8630429	+	1	H; M; R; C

^*a*^Some miRNA candidates had end variants. The sequence shown here was the most frequently identified in all the libraries. ^*b*^The total number of novel miRNA candidate in all sequenced clones.

^*c*^Conservation of bovine miRNA candidates was evaluated in human (Homo sapiens, H), mouse (Mus musculus, M), and rat (Rattus norvegicus, R), dog (Canis familiaris, C), horse (Equus caballus, E), chicken (Gallus gallus, G) genomes.]

*Indicating that the precursor of miRNA candidate was reported in bovine and other species as well as the other arm of the precursor was reported as miRNA.

To investigate evolutionary conservation of these miRNA candidates, six vertebrate genomes including human, mouse, rat, dog, horse and chicken were taken into account. Twelve out of 29 miRNA candidates were found to be conserved in at least one of these species. Three miRNA candidates (bta-un10, bta-un13 and bta-un20) were identified in the 5' arm of bta-mir-381, -495 and -487a, respectively, while only the 3' arms of these miRNAs were reported in several species (Table 2, Additional file 4).

### MicroRNA genes

A total of 207 genes encoding the identified bovine miRNAs were predicted using reported bovine miRNAs from miRBase 12.0 and the identified bovine orthologs of other mammalian miRNAs. Of these, 117 were reported in miRBase (version 12.0); 19 miRNA precursors were not reported in miRBase database previously; 71 were detected based on the orthologous miRNAs (Additional file 2). More than 30 bovine miRNAs were found to be encoded from more than one predicted hairpin precursor.

When the bovine miRNAs were compared to those from human, mouse and rat, most bovine mature miRNAs were also reported in the other three mammalian species. This agreed with that many miRNAs are conserved [17,18] among mammalian species. Most of the conserved miRNAs in the four species were also found to have the same number of precursor(s) although they were encoded by multi-copies of genes (For example, let-7a as shown in Additional file 5), indicating that the processing of these miRNAs has been conserved among these species.

### Sequence variations and end variants in mature miRNA sequences

**Figure 2:**
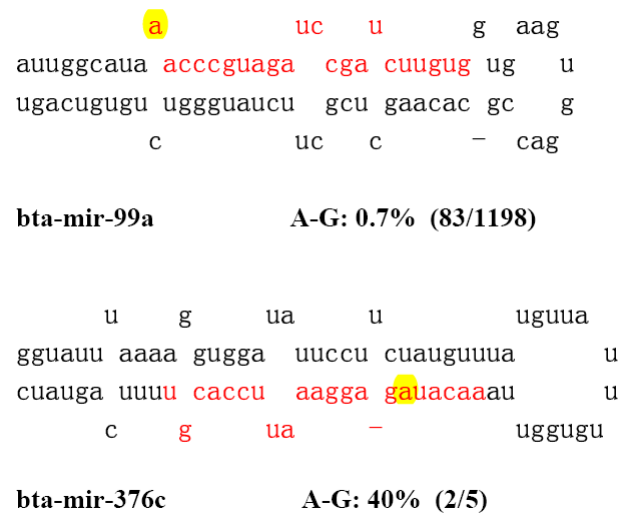
**Positions of edited adenosines in two bovine miRNAs**. Mature miRNA sequences are indicated in red letters. Edited adenosines are highlighted in yellow. The number of edited miRNA sequences and the number of all sequences of this miRNA are showed in brackets (edited/total).

Approximately 20% of the identified miRNA sequence were found to have mismatches with the bovine genome that were caused by post-transcriptional modification, and/or RT-PCR, and sequencing errors (Additional file 3). The prevailing sequence alterations such as 3' terminal A or U additions and A-to-G transitions could result from post-transcriptional modification as previously reported by Landgraf [13]. A-to-G transitions potentially caused by A-to-I editing were mainly identified in miR-99a and miR-376c (Figure 2), which have been reported to undergo A-to-I editing in human and mouse, respectively [22,23].

It has been reported that a variant of end (size) different siRNAs or miRNAs could be generated during Dicer processing of double-stranded RNA (dsRNA), short hairpin RNA (shRNA) and miRNA precursors *in vitro *[24,25]. Alignment of the identified sequences revealed that end variants were present in most miRNAs, showing that many miRNAs sequenced more than two times had end variants that were apparently generated from the same precursor (Additional file 3). Most end variants differed at the 3' end nucleotide(s) and a small percentage of them differed at the 5' end. The finding of the variant miRNA sequences is consistent with previous large scale miRNA profiling studies [13,14]. More than 10 bovine miRNAs reported in miRBase were one or two nucleotides shorter at the 3' end comparing to ours, for example, bta-miR-18a and bta-miR-16b (Additional files 2 &3).

### Analysis of miRNA expression in bovine tissues

**Figure 3:**
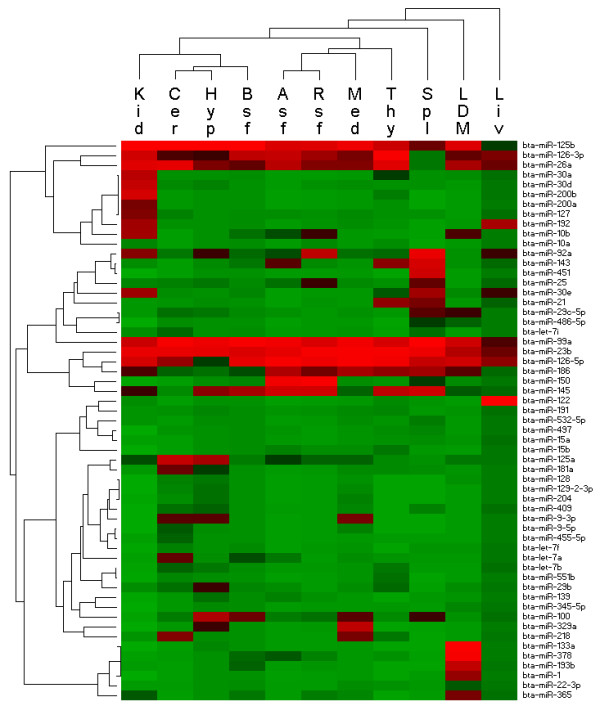
**Hierarchical clustering of tissues and miRNAs using Pearson correlation**. Heatmap was constructed based on the relative cloning frequencies of miRNAs. Only those miRNAs occuring at least 20 times in all libraries are shown. Green and red indicate low frequency or high frequency miRNA cloned in the library, respectively.

Hierarchical clustering of 11 bovine tissues based on the relative cloning frequencies of miRNAs showed that miRNA expression was similar in the related tissues such as abdominal subcutaneous fat and rump subcutaneous fat, while different tissues had diverse expression profiles (Figure 3). Comparison of miRNA expression profiles among tissues revealed that very few miRNAs expression was tissue specific (*e.g*., miR-9, -124 in brain, miR-122 in liver, miR-1, miR-133a and -206 in muscle). Several miRNAs were found to be highly expressed in particular tissues: miR-204, -218, and -129-2-3p in brain tissues, miR-30a, -30e, -30d, -200a and -200b in kidney, miR-192 in liver, miR-451 in spleen, miR-21 in spleen and thymus, miR-193b, -378 in LDM muscle. In contrast, 15 miRNAs were identified in all tissues and several of them (*e.g*., miR-23b and -99a) were expressed at high levels in all tissues. Another 31 miRNAs were found in 8 or more tissues. The five most abundant miRNAs across the 11 tissues were miR-23a, -23b, -99a, -125b and -126-5p, accounting for 44.3% of all small RNA sequences (Additional file 3).

**Figure 4:**
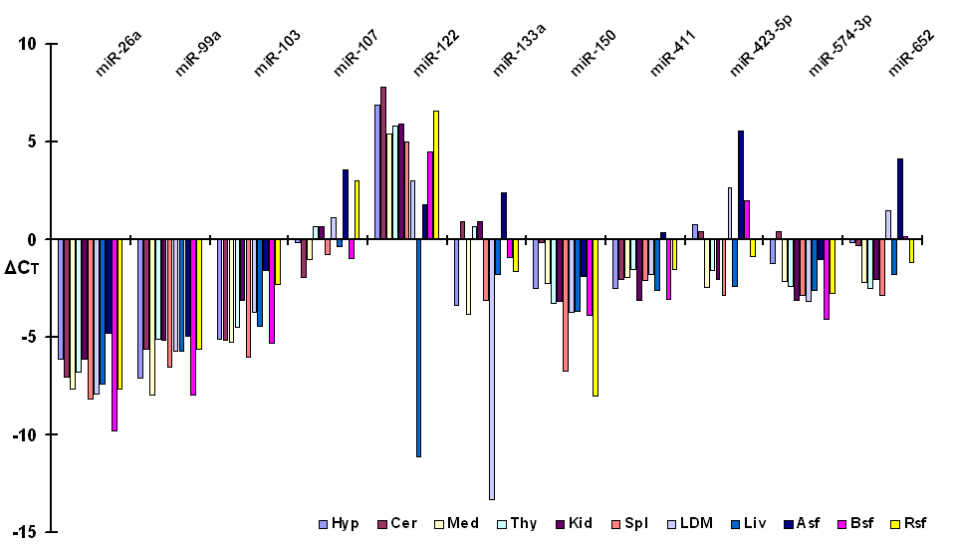
**Expression patterns of 11 miRNAs in 11 tissues. qRT-PCR results from 11 miRNAs across 11 tissues are compared**. miRNA expression profiles are normalized with RNU6B. High expressed miRNA has low ΔCt.

To validate above miRNA expression patterns, quantitative RT-PCR was performed on tissue-specific miRNAs (miR-122, -133a), high cloning frequency miRNAs (miR-26a, -99a and -150) and low cloning frequency miRNAs (miR-103, -107, -411, -423-5p, -574-3p and -652). Two animals were tested for each tissue and similar RT-PCR results were obtained from both animals (data not show). The expression profiles of the 11 miRNAs across 11 tissues confirmed that miR-26a and -99a expressed at high levels in all tissues, while miR-122 and -133a exclusively expressed in liver and muscle, respectively. Four bovine miRNAs (miR-411, -423-5p, -574-3p and -652) were expressed at low levels in all tissue (Figure 4). The qRT-PCR data showed similar expression trend of detected expression of these miRNAs from the library except miR-103.

## Discussion

MiRNA expression patterns in different tissues have been profiled in several vertebrates [13,26-30]. Although there were diverse expression profiles in different tissues, most miRNAs were ubiquitously expressed. Our comparison of miRNA expression across 11 tissues from bovine revealed a few tissue specific miRNAs: miR-9, -124 in brain, miR-122 in liver, miR-1, miR-133a and -206 in muscle, which had been previously reported in mouse and human [13,27]. Brain and muscle tissues have much more specific or enriched miRNAs than other tissues especially fat tissues, indicating that these miRNAs may play important regulatory roles in these tissues.

MiRNAs may be gained or lost during evolution, however, many miRNAs are conserved [17,18]. Identification of common miRNA precursors between bovine and other three mammalian species (human, mice, and rat) suggests these miRNAs may have similar roles as those in other species since these conserved miRNA have higher expression levels [14,31]. The higher expression level of three conserved miRNAs (miR-26a, -99a and -150) in all tested bovine tissues suggest that these miRNA may be more relevant to the highly conserved biological process in mammalians. Further study to discover their regulatory functions are needed. Recent developed hhigh-throughput sequencing analysis has allowed the identification of an increasing number of species-specific miRNAs [12,32] since these miRNA may play a role in host-specific biological process. Most of the miRNA candidates identified in this study were bovine specific, although their expression was less than 1% comparing the total sequenced clones, suggesting further studies using deep-sequencing technologies [12,14,32] or specialized small RNA isolation and cloning procedures [19,33] may help to identify and understand the functions of species specific miRNAs.

MiRNAs are firstly transcribed as pri-miRNAs and gave rise to short, 70-nucleotide stem-loop structures (pre-miRNAs) by the Drosha-DGCR8 complex [34,35]. The hairpin structures are then processed by Dicer. During the process, both strands of a miRNA precursor can form functional miRNAs. However, most of the time only one arm becomes the mature miRNA or predominant miRNA, while the other degrades or generates star miRNA. Khvorova et al [36] suggested that the arm with lower thermodynamic stability at its 5' end becomes the mature miRNA. In this study, we identified 15 pairs of bovine miRNAs and star miRNAs. The stability of the initial four base pairs of these miRNA pairs were calculated using nearest-neighbour method and 2-state hybridization algorithm [37,38]. Sixty percent of bovine miRNAs (9/15) displayed strand bias (Additional file 6). We also calculated the stability of human miRNAs and star miRNAs from miRBase 12.0 using the same method. In total, 175 pairs of miRNAs and star miRNAs were taken into account and 15 pairs of them were excluded from evaluation. Interestingly, only 60% (96/160) of human miRNAs exhibit strand bias, too (Additional file 7). We argue that there must be additional important factors other than internal stability to determine which arm of the miRNA precursor becomes the mature miRNA or miRNA* since the following observations can not be explained: (1) Most miRNAs observed in this study had a variant of isoforms generated by Dicer and a few of the 5' end variants even processed by Drosha (*e.g*., four bta-miR-23a variants had an additional A nucleotide at the 5' end, Additional file 3). (2) Some conserved pre-miRNAs express different mature miRNAs or star miRNAs depending on the species. For example, the stem-loop sequence of bta-miR-126 was perfectly matched to those from human, mouse and rat; however, in cattle and mouse, both strands were observed as mature miRNAs, while in human and rat, one strand generates miRNA and the other strand generates miRNA*. (3) Some mature miRNAs from the same precursors reverse to star miRNAs or vice versa in different tissues or development stage [39].

**Figure 5:**
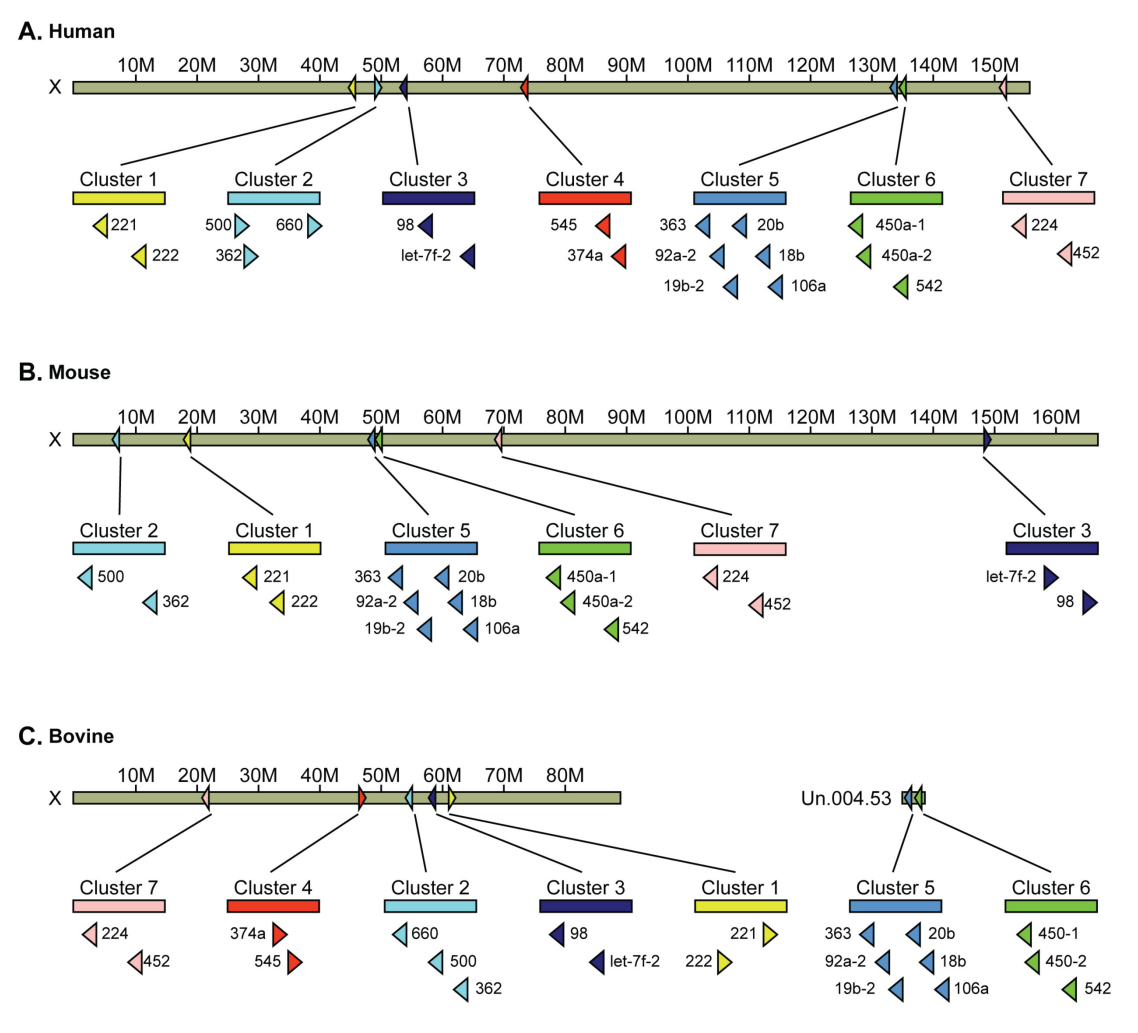
**Organization of X-linked miRNA Clusters in huaman (A), mouse (B) and bovine (C)**. Five to seven X-linked miRNA clusters contained ~20 miRNAs were identified from bovine, mouse and human, respectively. miRNA clusters are from ~250 bp (cluster2) to 4.9 kb (cluster5) in size. Cluster 4 was not identified in mouse. Arrow indicated the direction of transcription.

In addition, to understand the roles of miRNA, we mapped and identified Chromosome-X related miRNA. We identified five to seven miRNA clusters containing ~20 common miRNAs in bovine, mouse and human, respectively. Cluster 4 and the sequences of corresponding orthologs of these miRNAs were not found in mouse genome. We conjectured this cluster should be on some gap of chr.X in mouse. Two miRNA clusters (5 & 6) in bovine were not found on the X-chromosome but were found on the contig Un.004.53 (Figure 5, Additional file 8). Interestingly, another common miRNA (miR-652) was not mapped to the bovine genome but was also identified in the X-chromosome of human and mouse (Additional file 8). We speculate that contig Un.004.53 and bta-miR-652 belong to chromosome-X. The identification of miRNA and miRNA clusters on chromosome-X reveals that some miRNAs are conserved even in genome location between species. The BLAST search of the miRNAs on the bovine genome identified a third precursor, which was a perfect match with bta-mir-138-2 in contig Un.004.5037. However, only two precursors of miR-138 were found in human, rat and mouse. Conserved number copy of miRNA genes between species suggests that bta-mir-138-3 is the same as bta-mir-138-2 and contig Un.004.5037 may belong to chromosome 22. Therefore, miRNAs and their clustering appearance maybe provide potential molecular markers for evolution.

## Conclusion

Our small-RNA cloning and sequencing approach was efficiently target mature miRNAs. However, to identify and understand the functions of bovine miRNAs, further studies using deep-sequencing technologies are required. The miRNA expression patterns among 11 tissues in beef cattle showed most miRNAs are ubiquitously expressed, suggesting that these miRNAs may play a role in a broad range of biological processes in various tissues. Although mature miRNAs have been reported to display strand bias, the mechanism which arm of pre-miRNA dominates still need further study. Most identified bovine miRNAs were found in other three mammalian species and are highly conserved during evolution, suggesting that these miRNAs may have similar function in mammalian species and can be potential molecular markers for evolution.

## Methods

### Tissues collection and RNA extraction

Bovine tissues including longissimus dorsi muscle, kidney, liver, spleen, thymus, cerebellum, medulla, hypothalamus, abdominal subcutaneous fat and rump subcutaneous fat were collected from two 16-moth-old female Angus cross-breed cattle at the University of Alberta farm, while back subcutaneous fat tissue was collected from two 16-moth-old male Charolais cattle. Tissues were immediately frozen in liquid nitrogen and stored at -80°C until use. Total RNA and highly enriched small RNA (<200 nt) from tissues were extracted using mirVana miRNA Isolation Kit (Ambion Inc, Austin, TX, USA) according to the manufacturer's instruction.

### Small RNA library preparation and sequencing

Eleven cDNA libraries for small RNAs from the bovine tissues were constructed using miRCat™ Kit (IDT DNA Technologies, Coralville, IA, USA). Briefly, ~30 ug of enriched small RNA for each library was separated on 15% denatured polyacrylamide gels. The RNAs between 18 and 24 nt in size were recovered from the gels and ligated to the 3' linker using T4 RNA ligase. The ligated RNAs (~42 nt) were eluted from 15% denatured polyacrylamide gels and ligated to the 5' linker. The 5'-and 3' -linked RNAs (~62 nt) were reverse-transcribed to cDNAs. The cDNA products were amplified and the purified PCR products were digested by BanI and finally concatemerized and cloned into pCR2.1 TOPO vector using a TOPO cloning kit (Invitrogen, Carlsbad, CA, USA). The white colonies were randomly selected from the libraries and some of them were analyzed for inserts by PCR. The plasmids were extracted by MILLIPORE vacuum system and Montage Plasmid Miniprep Kit and sequenced by 3730 Sequencing Analyzer.

### Small RNA sequence analysis

A bioinformatics pipeline was developed for miRNA analysis. The small RNA sequences 18-26 in size were extracted and mapped to the latest bovine genome assembly (ucsc_btau4). All sequences were searched for miRNA sequences in miRBase. Small RNA sequence that had less than two mismatches (or >90% identity) with mammalian miRNAs in miRBase 12.0 was considered as a homologous miRNA and sorted by name without species prefix. Homologous miRNAs were further divided into previously reported bovine miRNAs and bovine orthologs of known miRNAs. Other small RNA sequences (mRNA, rRNA, tRNA, snoRNA, piRNA and misc RNA) were filtered by using BLAST against the NCBI nr database .

The genomic DNA sequences flanking the bovine miRNAs from miRBase 12.0, orthologs of known miRNAs and small RNA sequences mapped to the bovine genome but not aligned to any annotated RNA classes were obtained from the bovine genome assembly. Sequences were analyzed for hairpin structure using the UNAFold algorithm with default parameters or the Mfold web server at [40]. The hairpin structure containing putative miRNA was regarded as pre-miRNA if it contained neither large internal loops nor bulges, and at least 16 nt of the putative miRNA (if putative miRNA sequence length <22 nt, stretch 3' end to 22 nt) were paired with the other arm of the hairpin[18].

To investigate conservation of bovine novel miRNA candidates, highly similar DNA sequences corresponding to their precursors in the human, dog, mouse, rat, horse and chicken genome assemblies were found using BLATN NCBI GENOMES with word size = 7[41]. Sequence alignments covering > 70% identity and >75% of the length of precursors of bovine miRNA candidates were used for hairpin structure analyses as described above. The orthologs of bovine miRNA candidates were defined to be those who have proper hairpin structures and less than 3 nucleotides variation sequences with those in other species.

### Calculation of thermodynamic stability

Bovine miRNAs were sorted by clone counts. If the clone count of one strand was 10 times more than that of the other strand, they were regarded as miRNA and miRNA*, respectively. Human star miRNAs and their correspondingly high-expressed miRNAs were downloaded from miRBase (version 12.0). We used two-state hybridization web sever to calculate the free energy at . The internal stability of the initial four bases of miRNA or miRNA* (5'→3') were calculated as the delta G or delta G* (Additional files Table S5 & S6).

### Quantitative RT-PCR

Quantitative RT-PCR was performed using human TaqMan miRNA probes that had the same sequence as the bovine miRNAs. Reactions were performed following manufacturer's recommendations (Applied Biosystems, Foster City, CA, USA) except for 20 ng total RNA used.

### Heatmap construction

Hierarchical clustering of miRNA expression was performed with PermutMatrix [42], using Pearson distance, average linkage and normalized columns (Z score). Clustering was performed only on miRNA sequenced at least 20 times. The relative cloning frequency for each miRNA was calculated as the number of sequences for each miRNA in a library divided by the total number of miRNA sequences for that library.

## Abbreviations

Asf: abdominal subcutaneous fat; Bsf: back subcutaneous fat; Rsf: rump subcutaneous fat; Cer: cerebellum; Hyp: hypothalamus; Med: medulla; LDM: longissimus dorsi muscle; Kid: kidney; Liv: liver; Spl: spleen; Thy: thymus.

## Authors' contributions

LLG, and WJ designed the experiments; WJ performed library construction, sequence analysis, and original manuscript writing; JRG and PS developed bioinformatics pipeline for data analyses. SM involved in bovine genome mapping discussion and manuscript construction. WJ and LLG wrote the paper.

## Supplementary Material

Additional file 1 **Table S1- Description of 11 Small RNA libraries from bovine tissues**. miRNAs include reported bovine miRNAs and orthologs of known miRNAs from other mammalian species Click here for file

Additional file 2 **Table S2 - miRNAs and miRNA hairpins identified from bovine**. New bovine miRNAs identified in this study are filled in gray; miRNAs identified in this study but with a different size than those reported previous are filled in green; miRNAs not-mapped bovine genome and their hairpins listed here from human are filled in yellow. Click here for file

Additional file 3 **Table S3 - Distributions of miRNA and novel miRNA candidates in 11 bovine tissues**. miRNA sequences mismatched with their genome were regarded as modification (Md) if its 3' terminal has A or U additions, or the same site of miRNA, more than 2 sequences found A to G mutation (A-G transition). Other random mismatches were considered as mutations (Mt). Click here for file

Additional file 4 **Supplemental File S1 - Hairpin structures of 29 novel bovine miRNA candidates and their corresponding vertebrate orthologs**. Hairpin structure of novel bovine miRNA candidates and their corresponding vertebrate orthologs are listed together. Bovine miRNA candidates and their corresponding vertebrate orthologs are shown in uppercase. Variations between bovine miRNA candidates and vertebrate orthologs are indicated in red letters. For vertebrate orthologs, species (hsa, human; mmu, mouse; rno, rat; cfa, dog; eca, horse; gga, chicken), NCBI Accession Number, Genomic location are shown. Click here for file

Additional file 5 **Table S4 - Comparison of bovine miRNA genes with those from other three mammalian species**. 1: Genes for miRNA and related miRNA counted together are highlighted in yellow; "/" means "or". 2: All miRNA genes were from miRBase 12.0 and our study. 3: Star miRNAs were not listed here. Click here for file

Additional file 6 Table S5 - Thermodynamic stability of bovine miRNA and miRNA*. The internal stability of the initial four bases of miRNA or miRNA* (5'→3') were calculated as the delta G or delta G*, using two-state hybridization web sever () Click here for file

Additional file 7 Table S6 - Thermodynamic stability of human miRNA and miRNA*. The internal stability of the initial four bases of miRNA or miRNA* (5'→3') were calculated as the delta G or delta G*, using two-state hybridization web sever . Click here for file

Additional file 8 **Table S7 - X-linked miRNAs in bovine, mouse and human**. X-linked miRNAs from bovine, mouse and human, were clustered if they were less than 5,000 bp apart on the same strand. Click here for file
