# Supplementary material for: Characterization of bovine miRNAs by sequencing and bioinformatics analysis
Source: BMC Mol Biol. 2009 Sep 16;10:90. doi: 10.1186/1471-2199-10-90 (PMC2761914; doi:10.1186/1471-2199-10-90)
Supplement: Additional file 1 — Table S1- Description of 11 Small RNA libraries from bovine tissues. miRNAs include reported bovine miRNAs and orthologs of known miRNAs from other mammalian species [file 1471-2199-10-90-S1.pdf]

Table S1. Description of 11 Small RNA libraries from bovine tissues

Small RNA	Brain			Muscle	Subcutaneous Fat					Immune-related		
Sequence type	Cer	Hyp	Med	LDM	Asf	Bsf	Rsf	Liv	Kid	Spl	Thy	Total
miRNA	1117	925	1087	979	1318	1156	1351	1316	1162	1102	1243	12756
miRNA not mapped to bovine genome	2	1	0	1	1	0	0	1	0	0	5	11
novel miRNA candidates	6	8	6	1	10	5	13	16	8	6	23	102
piRNA	0	1	2	0	1	1	0	0	0	3	3	11
miscRNA	0	0	0	0	0	0	0	0	0	0	1	1
sn/sno-RNA	0	0	0	0	0	0	0	0	0	0	0	0
rRNA	24	32	102	41	27	33	11	48	38	38	12	406
tRNA	7	13	20	7	4	7	1	6	5	7	9	86
mRNA	2	11	3	8	8	4	4	4	10	16	12	82
not annotated-mapped to bovine genome	2	3	6	2	1	2	4	2	3	7	1	33
not annotated-not mapped to bovine genome	2	6	6	4	5	4	4	3	5	13	1	53
Overall squences	1162	1000	1232	1043	1375	1212	1388	1396	1231	1192	1310	13541
Other information												
enriched small RNA used (ug)	25	20	20	40	18	16	20	30	30	30	30	
clones sequenced	816	1032	1200	1920	1200	1008	1152	720	912	960	960	11880
clones contained small RNAs	527	518	693	757	681	556	666	536	567	493	558	6552
unique sequences	279	235	248	235	267	193	229	253	313	334	319	1420

miRNAs include reported bovine miRNAs and orthologs of known miRNAs from other mammalian species.
Tissues used for libraries construcation: abdominal subcutaneous fat (Asf); back subcutaneous fat (Bsf); rump subcutaneous fat (Rsf); cerebellum (Cer); hypothalamus (Hyp); medulla (Med); longissimus dorsi muscle (LDM); kidney (Kid); liver (Liv); spleen (Spl); thymus (Thy).
