# Supplementary material for: Characterization of bovine miRNAs by sequencing and bioinformatics analysis
Source: BMC Mol Biol. 2009 Sep 16;10:90. doi: 10.1186/1471-2199-10-90 (PMC2761914; doi:10.1186/1471-2199-10-90)
Supplement: Additional file 4 — Supplemental File S1 - Hairpin structures of 29 novel bovine miRNA candidates and their corresponding vertebrate orthologs. Hairpin structure of novel bovine miRNA candidates and their corresponding vertebrate orthologs are listed together. Bovine miRNA candidates and their corresponding vertebrate orthologs are shown in uppercase. Variations between bovine miRNA candidates and vertebrate orthologs are indicated in red letters. For vertebrate orthologs, species (hsa, human; mmu, mouse; rno, rat; cfa, dog; eca, horse; gga, chicken), NCBI Accession Number, Genomic location are shown. [file 1471-2199-10-90-S4.pdf]

Supplemental File S1

Hairpin structures of 29 novel bovine miRNA candidates and their corresponding vertebrate orthologs

```
01
>bta-un01-1 [-] chr16:7583033-7583128
ugagguauugaguuggucaaaaauuuuguugagguuuuucuguaagauguuauggAAAAACCUGAGUGAACUUUUCuggccaacucaauacuugga
..(((((((((((((((((((((.((..((.((((((((((((((....)))))))))))))).))..)).)))).)))))))))))))))))...    (-46.10)
>bta-un01-2 [+] chr27:21096060-21096155
aaaauuauugggucagccaaaauguucgcuuggcuuuuuccauacuaccuuacagAAAAACCUGAGUGAACUUUUCagcugauccaaugcuuccag
.....((((((((((((..(((.(((((((..(.((((((..((......))..)))))).)..))))))).)))..)))))))))))).......    (-34.80)


02
>bta-un02 [-] chrX:3009819-3009915
aaauaugugagugaaaaguuccgucaaccauccagcuguuugaggugaugcaaacAAACAUCUGGUUGGUUGAGAGAauuuuuuacuuggucucugg
........(((((((((((((..(((((((.((((.((((((..(.....)...)))))).)))).)))))))..))))))))))))).........    (-37.10)
>cfa; NC_006621; chrX:99628691-99628597
  auauuugagugaaaaguucagucaaccauccagcuguuugaguugaugcaagcAAACAUCUGGUUGGUUGAGAGAauuuuuuacuuagucucugg
  ....(((((((((((((((..(((((((.((((.((((((..(((...))).)))))).)))).)))))))..))))))))))))))).......    (-38.80)
>eca; NC_009175; chrX:99522669-99522573
aaaaaugugagugaaaaguucugucaaccauccagcuguuuggggugaugcaaacAAACAUCUAGUUGGUUGAGAGAauuuuuuacuuagucucugg
.......(((((((((((((((.(((((((...((.((((((..(.....)...)))))).))...))))))).)))))))))))))))........    (-35.90)


03
>bta-un03-1 [-] chr5:124162564-124162661
ugugucagaauuguguuggucaaaagcucauuggguuuuuuccgucacauacaggAAACCCGAACGAACUUUUGGGCCaacccaauagcauacgucug
(((((....((((.((((((((((((.((.((.(((((..(((..........)))))))).)).)).))))).))))))).)))).)))))......    (-30.90)
>bta-un03-2 [-] chr12:31995960-31996057
uugauuggguuggccaaaaugucuauuuggauuuuuccuuuacguccuauggaaaAAACCCGAACGAACUUUUGGGCCaacccaacauccacaaaaga
.((.(((((((((((.(((.((...(((((.(((((((............)))))))..)))))...)).))).)))))))))))))...........    (-32.50)
>bta-un03-3 [+] chr24:34630221-34630318
aaccguuaggugggccaaaaaauuuguuuggguuuuucuguaagaugguauggaaAAACCCGAACGAACUUUUGGGCCcaccuaauacauuuccacug
....((((((((((((.((((.((((((((((((((((((((......)))))))))))))))))))).)))).))))))))))))............    (-50.60)
>bta-un03-4 [+] chr24:42514990-42515087
gucuguauuggguuggcccaaaaguucauucgagcuuuuccuuaauauccuacggAAACCCGAACGAACUUUUGGGCCaacucaguacaucaguuaca
...((((((((((((((((((((((((.((((....(((((.((......)).)))))..)))).))))))))))))))))))))))))).........    (-48.10)
```

```
04
>bta-un04-1 [+] chr14:58301681-58301777
gaacuauuggguuggccaaaaaguuuguucagguuguuaguaaaaccguacagcuAAAGCUGAAUGAACUUUUUGGCcaaaccaguaagaauggaua
....(((((.((((((((((((((..(((((.(((((.(((......)))))..)).)))))..))))))))))))))).))))))..........    (-40.30)
>bta-un04-2 [+] chrX:72056992-72057088
gacagaacuauugguuggccagaaaguucauucagggcucuaacaucuuaugggaAAAGCUGAAUGAACUUUUUGGCcaaccaauaguucugucuuu
((((((((((((((((((((((((((((((((..(((..........))).....)))))))))))))))))))))))))))))))))...    (-62.00)
>bta-un04-3 [+] chrUn.004.21:291511-291607
auguauuggguuggccaacaaguucauuugggguuuucuguaccagcuucugguuAAAGCUGAAUGAACUUUUUGGCcaacccaaauagaaugaaga
.....(((((((((((.((((((((((..((((((....(((((...))))).)))))))))))))))).))))))))))))...........    (-42.80)


05
>bta-un05 [+] chr7:49895479-49895574
ugaguaccucugggcaguguUUUGCACCUCUGAGAGUGGAGuaacuccuccuguggaguugguccuagucugggugcaaacaguuuuuuucuauua
.(((.....(((........(((((((((...(((.((((.(((((((......))))))).))).).)))))))))))))))).....))).....    (-32.00)
>hsa; NC_000005.9; chr5:138614587-138614678
ugaguaccuuugggcaguguUUUGCACCUCUGAGAGUGGAAugacuccuguggaguugauccuagucugggugcaaacaauuuuuuucuguu
............(((((((((.((((((...(((.((((.(((((((...))))))).))).).))))))))))))))).......)))))    (-27.70)
>cfa; NC_006593; chr11:29760301-29760388
  aguacucuugggcagcguUUUGCACCUCUGAGAGUGGAAgaacuccuccuguggaguugguccuagucugggugcaaacaguuuuuuu
  .............(((..(((((((((...(((.((((..((((((......))))))..))).).))))))))))))..))).....    (-29.60)
>eca; NC_009157; chr14:37373449-37373354
ugaguaccuuugggcaguguUUUGCACCUCUGAGAGUGGAGuaacuccuccuguggaguugguccuagucugggugcaaacaauuuauuucuguua
............(((((((((.((((((...(((.((((.(((((((......))))))).))).).)))))))))))))))........))))).    (-33.70)



06
>bta-un06 [-] chr8:43938101-43938197
cguauuggguuggccagaaaguucauuugagguuuucuaucugaucuuacugaaaAACCCCAGAGAAACUUUCUGGCuaacccaauacaucaaacuc
.((((((((((((((((((((..((((.(((((((..............))))).)).))))..)))))))))))))))))))).........    (-47.20)


07
>bta-un07 [+] chrUn.004.13:739760-739855
uuucaauuauuggguuggccAAGAGUUUGUUCGGGUUUCUCcauaagcuguauagaaaaaccucaaugaacucuuuggccaacccaauauuuugcu
...(((.((((((((((((((((((..((.((((((.((.(((.....))).)).)))))).))..))))))).)))))))))))))..)))..    (-44.70)
```

08

>bta-un08 [-] chr21:65541215-65541311

cguccuggggcugccggaugAAGUACAGGAUGCCCAAUGAAUuaaauccaucuaaauacauuucacugggcauccucugcuuuauugggcugcccaa

.......((((.(((.(((((((((.((((((((((.((((....................)))).)))))))))).))))))))).))).))))..   (-49.20)

09

>bta-un09 [-] chr28:32693894-32693989

agacgggaggcagcagggccAGCCCUUCCCUUUCACUGGCCuguagccagcccaggucugguggga aggaagggacacauccugaugucugagugc

..((...(((((.(((((....(((((((.(..(((..((((..........)))).)..)))..).))))))).....))))).)))))..))..   (-42.40)

>eca; NC_009144; chr1: 6475309040-64752956

ggcagggccAGCCCUUCCCUGUCACUGGCCuauagccagcccagguuuggugggaagugagggacacguccugaugucugagugc

..((((((...(((((.((..((((((((.....)))))........)))..))....)))))....))))))............   (-28.30)

10

>bta-un10 [+] chr21:66021929-66022025 **(bta-mir-381)**

uacuaccguuugguacucagAGCGAGGUUGCCCUUUGUAUAUucgguuuuuugauguagaauauacaagggcaagcucucugugaguaucaaaccuu

.......((((((((((((.((.(((.((((((((.((((((((..............)))))))))))))))).))).)).))))))))))))...   (-48.10)

>hsa; NC_000014; chr14:101512251-101512340 **(hsa-mir-381)**

guuugguacuuaaAGCGAGGUUGCCCUUUGUAUAUucgguuuauugacauggaauauacaagggcaagcucucugugaguaucaaaccuu

((((((((((((.((.(((.((((((((.((((((((.((........)).)))))))))))))))).))).)).))))))))))))...   (-45.10)

>mmu; NC_000078; chr12:110965026-110965115 **(mmu-mir-381)**

guuugguacuuaaAGCGAGGUUGCCCUUUGUAUAUucgguuuauugacauggaauauacaagggcaagcucucugugaguaucaaaccuu

((((((((((((.((.(((.((((((((.((((((((.((........)).)))))))))))))))).))).)).))))))))))))...   (-45.10)

>rno; NC_005105; chr6:134407687-134407776 **(rno-mir-381)**

guuugguacuuaaAGCGAGGUUGCCCUUUGUAUAUucgguuuauugacaugggauauacaagggcaagcucucugugagcaucaaaccuu

(((((((.((((.((.(((.((((((((.((((((((.((........)).)))))))))))))))).))).)).)))).)))))))...   (-40.00)

>cfa; NC_006590; chr8:72317710-72317799 **(cfa-mir-381)**

guuugguacuuaaAGCGAGGUUGCCCUUUGUAUAUucgguuuauugacauagaauauacaagggcaagcucucugugaguaucaaaccuu

((((((((((((.((.(((.((((((.((((((((((.(((....)))...)))))))))))))))).))).)).))))))))))))...   (-44.20)

>eca; NC_009167; chr24:42916048-42916137

guuugguacuuaaAGCGAGGUUGCCCUUUGUAUAUucgguuuauugacauagaauauacaagggcaagcucucugugaguaucaaaccuu

((((((((((((.((.(((.((((((.((((((((((.(((....)))...)))))))))))))))).))).)).))))))))))))...   (-44.20)

11

>bta-un11 [+] chr1:76369830-76369927

gcgcgucgcagggacccgcgCAGUCCGGUCCCGCGGUGUCUCCgccuguugaugccgcgcggcccgcgggcucggaugcucggcgcucugcccuggca

((.((..((((((..(((.(((.((((..(((((((.(((..(((..((....)).))).))))))))))..))))))).)))..))))))..)))).   (-52.50)

```
12
>bta-un12 [-] chrX:21829420-21829516
auacccaaugaaugcgaaaaCCUCAGUCAGCCUUGUGGAUGUauguucugcagaccugacaucuagaggacugacugaaauuuucacuuucagcuaa
........(((...(((((..(((((((((((.(((((((..(((.....)))...))))))))))).))))))))..)))))...))))).....    (-32.30)
>hsa; NC_000023; chrX:151125246-151125155
   ccaagugacugugaggaCCUCAGUCUGCCCUGUGGAUGUguaugccacagaccugacuucuggagggcugacugaaauguucauuuuaagcu
   ...(((....(((((....(((((.(((((.((((((((.....))))........)))).))))).))))))....)))))).....)))    (-28.20)
>eca; NC_009175; chrX:120686799-120686702
ugcccaagugaaugugaagaCCUCGGUCUGCCUUGUGUAUGUauacucugcagaccugauaucuggagcgcuggcugaaauguucacuuucaacuaa
.....(((((((((..(((..(((((((((...((((....))))...))))))).))..)))..((((....)))...))))))))))........    (-26.33)


13
>bta-un13 [+] chr21:66012099-66012195                                        (bta-mir-495)
ugaccacuggugccuggaaaGAAGUUGCCCAUGUUCUUUUCGcugauaugugacgaaacaaacauggugcacuucuuuuccggcaucaaaucucagc
(((....(((((((.((((((((((.(((((((((..(((((..........)))))..))))))).))))))))))))).)))))))....)))..    (-40.80)
>hsa; NC_000014; chr14:101500091-101500179                                    (hsa-mir-495)
     cugguaccugaaaaGAAGUUGCCCAUGUUAUUUUCGcuuuauaugugacgaaacaaacauggugcacuucuuuuucgguaucaaauauc
     .(((((((.((((((((((.(((((((((..(((((.((.......)))))))..))))))).))))))))))))).))))))).......    (-35.80)
>mmu; NC_00007812; 110956954-110957044                                        (mmu-mir-495)
      ugguaccugaaaaGAAGUUGCCCAUGUUAUUUUUCGcuuuuauuugugacgaaacaaacauggugcacuucuuuuucgauaucaaaucuca
      (((((...((((((((.((((((((((.(((.((((........)))).)))...))))))).))))))))))))...)))))........    (-28.90)
>rno; NC_005105; chr6:134399215- 134399305                                    (rno-mir-495)
       ugguaccugaaaaGAAGUUGCCCAUGUUAUUUUUCGcuuuuauuugugacgaaacaaacauggugcacuucuuuuucgauaucaaaacuca
       (((((...((((((((.((((((((((.(((.((((........)))).)))...))))))).))))))))))))...)))))........    (-28.90)
>cfa; NC_006590; chr8:72307884-72307976                                      (cfa-mir-495)
    accucuggugccugggaaGAAGUUGCCCGUGUUAUUUUCGcuugauacgugccgaaacaaacauggugcacuucuuucucgguaucaaaucuc
    .....(((((((.(((((((((.((((((((((..((((((.........)).))))..))))))).))))))))))))).))))))).......    (-37.70)
>eca; NC_009167; chr24:42906083-42906172
cuggucccugaaaaGAAGUUGCCCAUGUUAUUUUCGcuugauaugugacgaaacaaacauggugcacuucuuuuucgguaucaaaucuca
      .((((.((.((((((((((.(((((((((..(((((.((.......)))))))..))))))).)))))))))))).)).)))).......    (-31.10)
```

14

>bta-un14 [-] chr1:65888797-65888892

augagaaaaccuguucucuuGAGAGAUCAGAGGCGCAGAGUgcgucaaugucaaugaagccugugccuuuuaccucuuuaagagcgcacgguuuca

....(.((((((((.((((((((((...((((((((((...((.(((.......))).))))))))))))...))))).))))).))).)))))).   (-36.30)

>hsa; NC_000003; chr3:119803502-119803407

augagaaaaccuguucucuuGAGAGAUCAGAGGCGCAGAGUgcgucagugucaaugaagccugugccuuuuacuucuuuaagagcguacgguuuca

....((((.((((..((((((((((...((((((((((...((.(((.......))).))))))))))))...))).)))))))..)).)))))).   (-31.90)

>cfa; NC_006615; chr33:26689455-26689359

augagaaaaaccuguucucuuGAGAGAUCAGAGGCGCAGAGUgcgucaaugucaaugaagccugugccuuuuaccucuuuaagagcgcacgguuuca

.......((((((((.((((((((((...((((((((((...((.(((.......))).))))))))))))...))))).))))).))).)))))..   (-35.70)

>eca; NC_009167; chr19: 38814104-38814199

augagacaaccuguucucuuGAGAGAUCAGAGGCGCAGAGUgcgucaaugucagugaagccugugccuuuuaccucuuuaagagcgcacgguuuca

....((.(((((((.((((((((((...((((((((((...((.(((.......))).))))))))))))...))).))))))).))).)))))).   (-36.30)

15

>bta-un15 [+] chr1:76369833-76369930

cgucgcagggacccgcgcaguccggucccgcggugucuccgccuguugaugccgcGCGGCCCGCGGGCUCGGAUGCUcggcgcucugcccuggcagcg

.(((((((((..(((.(((.((((..(((((((.(((..(((..((....)).))).))))))))))..))))))).)))..))))))...)))....   (-51.20)

16

>bta-un16 [+] chr26:31723009-31723105

cuauuaacaaggacaccaguuaggccacucagaguaacuaucaguaacuaacuggUUACUCUGAGUAACCUAACUGguauccuuguuauuagucauc

(((.(((((((((.(((((((((..(((((((((((((...((((.....))))))))))))))))))..)))))))))).))))))))).))).....   (-54.00)

17

>bta-un17-1 [+] chrX:18300090-18300185

cuacucauuccaguauucuuGGACUUCCCUGGUAGCUCAGaugguaaagaauccaccugcagugcggaagaucugggguucaaucccugguugga

...((((..((((....((..(((((((...(((.(((((.(((........))).))).)))))))))).))..))........))))..)))).   (-29.60)

>bta-un17-2 [-] chrX:82012934-82013029

acuuccuauucccuggggauuGGACUUCCCUGGUAGCUCAGaugguaaagcgucugccugcaaugcaggagaccugguucgaucccuggucagga

...((((...(((.((((((((((((((((((((((..((((((......)))))).))))....)))).))...)))))))))))))))..))))   (-41.80)

18

>bta-un18 [+] chr21:66028337-66028433

uccgugauaggggaguggauGGUUGAUCAGAGAACAUACAUUuugucaaugauguaugucaacugauccacagucccucccuauccagucacuucuc

...(((((.((((((.((((.((.((((((...(((((((((........)))))))))...)))))).)).))))))))))).....)))))).....   (-40.00)

>hsa; NC_000014; chr14:101518341-101518436

uccaugauaggggaauggauGGUUGAUCAGAGAACGUACAUUuuauaaaugguguaugucaguugauccacagucccuaccuauccagugacuucu

..(((((((((.....((((.((.((((((...(((((((((........)))))))))...)))))).)).))))...))))))..)))......   (-30.70)

>cfa; NC_006590; chr8:72323381-72323477
uccaugauaggggaauggauGGUUGAUCAGAGAACAUACAUUuuaucaaugauguaugucaacugauccacagucccuaccuauccagugacuucuc
..((((((((.....((((.((.((((((...((((((((........))))))))...)))))).)).))))...))))))..))).......   (-32.70)
>eca; NC_009167; chr24:42921551-42921647
uccaugauaggggaauggauGGUUGAUCAGAGAACAUACAUUuuaucaaugauguaugucaacugauccacagucccuaccuauccagagacuucuc
.....((((((.....((((.((.((((((...((((((((........))))))))...)))))).)).))))...))))))..(((....)))   (-33.10)

19
>bta-un19 [+] chr7:42028168-42028262
gcgcaucagccugucgcagaaagcaggccguucaggaacauccucccuucagcguGUCAGGAUGGCCGAGUGGUCuaaggcgccagacucaaggu
..((...((.(((.((((((..((.((((((((..((.(((.((......)).))))).))))))))..))..)))...))).))).))....))   (-27.10)

20
>bta-un20 [+] chr21:66028725-66028821 **(bta-mir-487a)**
uagccuuugguacuugaagaGUGGUUAUCCCUGCUGUGUUCGcuguauuuaugacgaaucauacagggacauccaguuuuucaguaucaaauacugc
(((..(((((((((.(((((.(((...((((((.((((((((..((....))..)))).))))))))))...))).))))).)))))))))..))).   (-36.30)
>hsa; NC_000014; chr14:101518776-101518871 **(hsa-mir-487a)**
agccuucgguacuugaagaGUGGUUAUCCCUGCUGUGUUCGcuuaauuuaugacgaaucauacagggacauccaguuuuucaguaucaaauacugc
.......((((((.(((((.(((...((((((.((((((((.(((.....))))))).))))))))))...))).))))).))))))........   (-32.30)
>cfa; NC_006590; chr8:72323803-72323899 **(cfa-mir-487a)**
uagccuucgguacuugaagaGUGGUUAUCCCUGCUGUGUUCGcuguauuuaugacgaaucauacagggacauccaguuuuucaguaucaaauacugc
........((((((.(((((.(((...((((((.((((((((..((....))..)))).))))))))))...))).))))).))))))........   (-32.90)
>eca; NC_009167; chr24:42921984-42922080
uagccuucgguacuugaagaGUGGUUAUCCCUGCUGUGUUCGcuguauuuaugacgaaucauacagggacauccaguuuuucaguaucaaauacugc
........((((((.(((((.(((...((((((.((((((((..((....))..)))).))))))))))...))).))))).))))))........   (-32.90)
>mmu; NC_000078; chr12:110965540-110965625 **(mmu-mir-487b)**
cuuggguacuuggagaGUGGUUAUCCCUGUCCUCUUCGcuucacucaugccgaaucguacagggucauccacuuuuucaguaucaa
..((((((((..((((((((....((((((....((((.........)).)))....))))))....))))))))..))))))))   (-32.40)
>rno; NC_005105chr6:134408206-134408291 **(rno-mir-487b)**
cuuggguacuuggagaGUGGUUAUCCCUGUCCUCUUCGcuucacucaugccgaaucguacagggucauccacuuuuucaguaucaa
..((((((((..((((((((....((((((....((((.........)).)))....))))))....))))))))..))))))))   (-32.40)

21
>bta-un21 [+] chr21:68561312-68561409
cccgggagggggccggggcgcgcagggagggggcggcuggcuuuguccgagcccgUCCCCUUCCUUCCGGCCUCCGCCcucucccgaggcaccgcccu
..((((((((((.(((((.((.(((((((((((((((((......)).)))).)))))))))))..))))))).))))))))))).(((...)))..   (-68.30)

```
22
>bta-un22 [+] chr11:102046747-102046842
gcuggccuuugagcucacugcggggugggcauggcuaagggaucccugggaacccUCCGCCUUGUGCUUCCUGCAgauccugcaccgggacaugaa
(((........)))...((((((((..((((((((..((((.(((...))).))))..)))..)))))))))))))).(((((...)))))......      (-38.00)


23
>bta-un23 [-] chr1:3473974-3474069
ggauuuaguauugguuggcUGAAAAGUUCGUUCGGGUUUUuccauaagaacucaaauaaacuuuuuggccaacccaguaauaacacugaugcacc
....(((.((((((((((((..(((((((..((.((((((((....)))))))).))..)))))))..)))))))))))).))).........      (-39.80)


24
>bta-un24-1 [-] chr21:55700328-55700424
cuucgaucuuugucauggugUGCGGGAUCUUUAGUUGUGGUGugcaaacucucaguugcagcuugugggauccaguuccaugaccaaggauuggacc
.((((((((((((((((...((.(((((((.(((((..(((..........)).)..)))))...))))))).)).))))))).)))))))))..      (-36.50)
>bta-un24-2 [-] chr25:35972410-35972506
aaacagccgacguugcggcaUGCGGGAUCUUUAGUUGUGGUGugugaauucuuaguugcagcaacuaaagaucccucaggcugcaaugaagaucaga
..........((((((((.((.(((((((((((((((.(((..(((....)))..))).))))))))))))))).)).))))))))).........      (-42.50)



25
>bta-un25 [-] chr7:5546135-5546232
gcaaggugucgggguggggggUGGGGGGAGGCCACACCAUGUCAcugcagggcuccucgcugacugggguuggccccaucccaggucgguuccuguggcu
.......(((((..(.((..(((((((.(((((..(((.((((..((..(....)..)))))))))..))))))).)))))..)).)..))...))).      (-41.00)



26
>bta-un26-1 [-] chr10:66855635-66855731
auagugguuaguacucugcgUUGUGGCCGCAGCAACCUCGGUucgaauccgagucacggcauuguggcaacaauggcacggcaggggcgucuuuuuu
..........((.(((((((((((.((((((((...(((((.......))))).....))..)))))).))))).....)))))))).........      (-30.30)
>bta-un26-2 [+] chr10:66857949-66858045
auagugguuaguacucugcgUUGUGGCCGCAGCAACCUCGGUucgaauccgagucacggcauuguggcaacaauggcacggcaagggaccucuacuu
.(((.((((....((.((((((((.((((((((...(((((.......))))).....))..)))))).)))))).)).)).....)))).)))...      (-33.70)
>hsa; NC_000015; chr15:45493360-45493451
auagugguuaguacucugcgUUGUGGCCGCAGCAACCUCGGUucgaauccgagucacggcauuguggaaacaaugguacggcaagggccucu
..((.((((....((.((((((((..(((((((...(((((.......))))).....))..)))))..)))))).)).))....)))).))      (-27.90)
>hsa; NC_000015; chr15:45490864-45490778
auagugguuaguacucugcgUUGUGGCCGCAGCAACCUCGGUucgaauccgagucacggcauugugggaacaauggcacggcaaggg
.............((.((((((((..(((((((...(((((.......))))).....))..)))))..)))))).)).)).......      (-23.30)
```

```
>mmu; NC_000068; chr2:122203715-122203810
auagugguuaguacucugcgUUGUGGCCGCAGCAACCUCGGUucgaauccgagucacggcauuguggcaacaugguacggcaagggcgccuuu
..........((((....((((((.((((((((...(((((.......))))).....))..)))))).)))))))))))(((......)))...        (-30.60)
>mmu; NC_000068; chr2:122201290-122201204;122203159-122203073
auagugguuaguacucugcgUUGUGGCCGCAGCAACCUCGGUucgaauccgagucacggcauuguggcaacaauggcacggcaaggg
............((.((((((((.((((((((...(((((.......))))).....))..)))))).)))))).)).))......        (-26.30)
>rno; NC_005102; chr3:109164053-109164148
auagugguuaguacucugcgUUGUGGCCGCAGCAACCUCGGUucgaauccgagucacggcauuguggcaacaauggcacggcaaagggcgcuuuuu
..((((.((....((.((((((((.((((((((...(((((.......))))).....))..)))))).)))))).)).))....)).))))....        (-29.70)
>rno; NC_005102; chr3:10916954-109169459; 109171051-109170965; 109413554-109413640
auagugguuaguacucugcgUUGUGGCCGCAGCAACCUCGGUucgaauccgagucacggcauuguggcaacaauggcacggcaaggg
............((.((((((((.((((((((...(((((.......))))).....))..)))))).)))))).)).))......        (-26.30)
>cfa; NC_006612; chr30:14607221-14607135; 14609442-14609528
auagugguuaguacucugcgUUGUGGCCGCAGCAACCUCGGUucgaauccgagucacggcauugugggaacaauggcacggcaaggg
............((.((((((((..(((((((...(((((.......))))).....))..)))))..)))))).)).))......        (-23.30)
>eca; NC_009144; chr1:144233980-144233885
auagugguuaguacucugcgUUGUGGCCGCAGCAACCUCGGUucgaauccgagucacggcauugugggaacaauggcacggcaagggcgccuuuuu
.................((((((..(((((((...(((((.......))))).....))..)))))..)))))).....(((......))).....        (-26.10)
```

27

```
>bta-un27-1 [-] chr12:14082778-14082867
cgucgucucccacauggucuagcgguuaggauuccugguuuucacccaggcggcccgggUUCGACUCCCGGUGUGGGAACgccaccaccu
.(.(((.((((((((((((.(((..(..((...(((((.......)))))...))..)))).))))....))))))))))))........        (-29.20)
>bta-un27-2 [+] chrUn.004.4221:9612-9703
cgucgucucccacauggucuagcgguuaggauuccugguuuucacccaggcggcccgggUUCGACUCCCGGUGUGGGAACgccaccaccuuu
.(.(((.((((((((((((.(((..(..((...(((((.......)))))...))..)))).))))....))))))))))))..........        (-29.20)
>hsa; NC_000013; chr13:45492137-45492058
  cgucucccacauggucuagcgguuaggauuccugguuuucacccaggcggcccgggUUCGACUCCCGGUGUGGGAACgcc
  (((.((((((((((((.(((..(..((...(((((.......)))))...))..)))).))))....))))))))))).. (-28.90)
>hsa; NC_000013; chr15:26327458-26327368
gucaucucccacauggucuagcgguuaggauuccugguuuucacccaggcggcccgggUUCGACUCCCGGUGUGGGAAUgacuggaccuuu
(((((.((((((((((((.(((..(..((...(((((.......)))))...))..)))).))))....)))))))))))))).........        (-32.90)
>mmu; NC_000080; chr14:76552575-76552657
gucgucucccacauggucuagcgguuaggauuccugguuuucacccaggcggcccgggUUCGACUCCCGGUGUGGGAACgcca
..(((.((((((((((((.(((..(..((...(((((.......)))))...))..)))).))))....)))))))))))...        (-29.00)
>mmu; NC_000075; chr9:104311068-104310996
      ucccacauggucuagcgguuaggauuccugguuuucacccaggcggcccgggUUCGACUCCCGGUGUGGGAAC
      (((((((((((.(((..(..((...(((((.......)))))...))..)))).))))....)))))))))..        (-27.10)
>mmu; NC_000073; chr7:65654685-65654757
       ucccacauggucuagcgguuaggauuccugguuuucacccaggcggcccgggUUCGACUCCCGGUGUGGGAAC
(((((((((((.(((..(..((...(((((.......)))))...))..)))).))))....)))))))))..        (-27.10)
```

```
>rno; NC_005114; chr15:57083842-57083924
  gucgucucccacauggucuagcgguuaggauuccugguuuucacccaggcggcccgggUUCGACUCCCGGUGUGGGAACgcca
  ..(((.((((((((((((.(((..(..((...(((((.......)))))...))..)))).)))).....)))))))))))...                         (-29.00)
>rno; NC_005114; chr15: 61543594-61543666
        ucccauauggucuagcgguuaggauuccugguuuucacccaggcggcccgggUUCGACUCCCGGUAUGGGAAC
        ((((((((((((.(((..(..((...(((((.......)))))...))..)))).)))).....))))))))..                         (-25.20)
>cfa; NC_006604; chr22:9049413-9049496
 cgucgucucccacauggucuagcgguuaggauuccugguuuucacccaggcggcccgggUUCGACUCCCGGUGUGGGAACgcca
 .(.(((.((((((((((((.(((..(..((...(((((.......)))))...))..)))).)))).....))))))))))))..                         (-29.20)
>cfa; NC_006585; chr3:37423794-37423874
  gucgccucccauauggucuagcgguuaggauuccugguuuucacccaggcggcccgggUUCGACUCCCGGUGUGGGAAUgc
  ...((.((((((((((((.(((..(..((...(((((.......)))))...))..)))).)))).....))))))))..))                         (-25.60)
>eca; NC_009160; chr17:25605879-25605959
   gucgucucccacauggucuagcgguuaggauuccugguuuucacccaggcggcccgggUUCGACUCCCGGUGUGGGAACgc
   ..(((.((((((((((((.(((..(..((...(((((.......)))))...))..)))).)))).....))))))))))).                         (-29.00)
>eca; chr1:111509436-111509357
    gucgucucccacauggucuagcgguuaggauuccugguuuucacccaggcggcccgggUUCGACUCCCGGUGUGGGAAGa
    ......((((((((((((.(((..(..((...(((((.......)))))...))..)))).)))).....))))))))...                         (-27.20)
>gga; NC_006107; chr20:992599-992673
          ucccacauggucuagcgguuaggauuccugguuuucacccaggcggcccgggUUCGACUCCCGGUGUGGGAAAgc
          ((((((((((((.(((..(..((...(((((.......)))))...))..)))).)))).....))))))))....                         (-27.10)
```

```
28
>bta-un28 [+] chr4:106953575-106953673
ggcuacccacuccaguauucGUGGACUUCCCUGGUAGCUCAGCUgguaaagaauccgcuugcaacgugggaauacuagauucgaucccugguugggaa
.....((((..((((...((((((..(((((..((.((..(((.((........))))).)).))..)))))..)))....)))...))))..))))..  (-30.60)
```

```
29
>bta-un29 [+] chr3:8630334-8630435
cgccggggcucgcgccgcgucggguccccagccaggcgagcggccacgcgcccagccaggcccgccGUCAGGAUGGCCGAGCGGUCUaaggcg
((((..((.....(((((.((((...(((.((.((((.((((((..((.....))..)).)))))))).)).))))))))))))))..))))      (-43.80)
>hsa; NC_000001; chr1:161418695-161418767; 161426076-161426148; 161433486-161433558;
                      161440867-161440939; 161411277-161411349
                   cgcgccggguccccagccaugccagcggcgacgcgccccggcgcgccGUCAGGAUGGCCGAGCGGUCUaaggcg
                   ..((((.((.((..(((((.((.((((((.(((......)))))))))..))))))).....)).))..))))      (-33.70)
>mmu; NC_000067; chr1:172996069-172996141;173003704-173003776;173011318-173011390
                   gggcgccggacgccugcgaggcgagugagcuggagcccaaaccgccGUCAGGAUGGCCGAGCGGUCUaaggcg
                   ((.((((((.((((((...((((..((.((....)).))...))))..)))).)).))).))).)).......      (-29.20)
>rno; NC_005112; chr13:86914705-86914782; 86934528-86934605;86956756-86956833
              cgccggucgccggucgccugcgaggcgagcggucuacgccccagaccugcuGUCAGGAUGGCCGAGCGGUCUaaggcg
              (((((..(((((((((((((...((((...(((((.......)))))))))..)))).)))))).)))..)...))))      (-38.30)
>cfa; NC_006620; chr38:24093788-24093717; 24108623- 24108552
                    gcgccggguccccagccaggcgagcggcggcgcgcccaggcccgccGUCAGGAUGGCCGAGCGGUCUaaggcg
                    .((((.((.((..((..(((....(((((((.((....)).))))))).......)))..)))).))..))))      (-32.20)
```
