# Supplementary material for: Characterization of bovine miRNAs by sequencing and bioinformatics analysis
Source: BMC Mol Biol. 2009 Sep 16;10:90. doi: 10.1186/1471-2199-10-90 (PMC2761914; doi:10.1186/1471-2199-10-90)
Supplement: Additional file 8 — Table S7 - X-linked miRNAs in bovine, mouse and human. X-linked miRNAs from bovine, mouse and human, were clustered if they were less than 5,000 bp apart on the same strand. [file 1471-2199-10-90-S8.pdf]

Table S7. X-linked miRNAs in bovine, mouse and human

Cluster	Bovine [a]						Human [b]					Mouse [c]				
	pre-miRNA	Count	Chr./ Co.	Start	End	strand	pre-miRNA	Chr.	Start	End	strand	pre-miRNA	Chr.	Start	End	strand
1	bta-mir-221	19	X	61412746	61412855	+	hsa-mir-221	X	45490529	45490638	-	mmu-mir-221	X	18723420	18723514	-
	bta-mir-222	5	X	61412052	61412161	+	hsa-mir-222	X	45491365	45491474	-	mmu-mir-222	X	18724019	18724097	-
	bta-mir-532	14/32	X	54783137	54783222	-	hsa-mir-532	X	49654494	49654584	+	mmu-mir-532	X	6825528	6825623	-
2	bta-mir-500	4/7	X	54769936	54770033	-	hsa-mir-500	X	49659779	49659862	+	mmu-mir-500	X	6814809	6814900	-
	bta-mir-362	11	X	54771199	54771297	-	hsa-mir-362	X	49660312	49660376	+	mmu-mir-362	X	6819108	6819172	-
	bta-mir-660	1	X	54767794	54767873	-	hsa-mir-660	X	49664589	49664685	+		gap?			
3	bta-mir-98	0	X	58737622	58737740	-	hsa-mir-98	X	53599909	53600027	-	mmu-mir-98	X	148347757	148347864	+
	bta-let-7f-2	11.5	X	58738526	58738608	-	has-let-7f-2	X	53600878	53600960	-	mmu-let-7f-2	X	148346889	148346971	+
	bta-mir-1468	5	X	58411908	58412004	+	hsa-mir-1468	X	62922607	62922692	-		gap?			
	bta-mir-223	2	X	56723507	56723603	-	hsa-mir-223	X	65155437	65155546	+	mmu-mir-223	X	93438156	93438265	+
	bta-mir-374b	14	X	46943043	46943140	+	hsa-mir-374b	X	73355107	73355178	-	mmu-mir-374	X	100768399	100768493	-
4	bta-mir-545	0	X	46871699	46871804	+	hsa-mir-545	X	73423664	73423769	-		gap?			
	bta-mir-374a	14	X	46871549	46871620	+	hsa-mir-374a	X	73423846	73423917	-		gap?			
	bta-mir-361	1/3	X	42524578	42524648	+	hsa-mir-361	X	85045297	85045368	-	mmu-mir-361	X	110188433	110188502	-
	bta-mir-652	9	gap				hsa-mir-652	X	109185213	109185310	+	mmu-mir-652	X	139173543	139173640	+
5	bta-mir-363	1	Un.004.53	180833	180906	-	hsa-mir-363	X	133131074	133131148	-	mmu-mir-363	X	50094870	50094944	-
	bta-mir-92a-2	138	Un.004.53	180988	181055	-	hsa-mir-92a-2	X	133131234	133131308	-	mmu-mir-92a-2	X	50095015	50095105	-
	bta-mir-19b-2	3.5	Un.004.53	181139	181221	-	hsa-mir-19b-2	X	133131367	133131462	-	mmu-mir-19b-2	X	50095160	50095243	-
	bta-mir-20b	1	Un.004.53	181264	181332	-	hsa-mir-20b	X	133131505	133131573	-	mmu-mir-20b	X	50095290	50095369	-
	bta-mir-18b	0	Un.004.53	181499	181573	-	hsa-mir-18b	X	133131737	133131807	-	mmu-mir-18b	X	50095508	50095590	-
	bta-mir-106a	0	Un.004.53	181658	181738	-	hsa-mir-106a	X	133131894	133131974	-	mmu-mir-106a	X	50095680	50095744	-
6	bta-mir-450-1	3	Un.004.53	440487	440729	-	hsa-mir-450a-1	X	133502037	133502127	-	mmu-mir-450a-1	X	50401331	50401421	-
	bta-mir-450-2	3	Un.004.53	440638	441646	-	hsa-mir-450a-2	X	133502204	133502303	-	mmu-mir-450a-2	X	50401476	50401544	-
	bta-mir-542	1	Un.004.53	441550	440568	-	hsa-mir-542	X	133503037	133503133	-	mmu-mir-542	X	50402580	50402664	-
	bta-mir-504	6	X	12002347	12002444	-	hsa-mir-504	X	137577538	137577620	-	mmu-mir-504	X	56350835	56350913	-
	bta-mir-505	4	X	13241304	13241399	+	hsa-mir-505	X	138833973	138834056	-	mmu-mir-505	X	57647578	57647667	-
7	bta-mir-224	4	X	21831345	21831443	-	hsa-mir-224	X	150877706	150877786	-	mmu-mir-224	X	69506370	69506451	-
	bta-mir-452	1	X	21832378	21832472	-	hsa-mir-452	X	150878756	150878840	-	mmu-mir-452	X	69507563	69507647	-

X-linked miRNAs from bovine, mouse and human, were clustered if they were less than 5,000 bp apart on the same strand.

a: miRNA counts for sequences that matched two or more different precursors are split equally among them. Mature miRNAs generated from the same precursor are listed and split with "/".

b: Precursors of miRNA sorted according to the position of human miRNAs.

c: Pre-miRNAs unidentified in mouse X chromosome may be result of gaps there.
